# Does Domestication Affect Structural and Functional Leaf Epidermal Traits? A Comparison between Wild and Cultivated Mexican Chili Peppers (*Capsicum annuum*)

**DOI:** 10.3390/plants11223062

**Published:** 2022-11-11

**Authors:** Carlos Serrano-Mejía, Rafael Bello-Bedoy, María Clara Arteaga, Guillermo R. Castillo

**Affiliations:** 1 Facultad de Ciencias, Universidad Autónoma de Baja California, Carretera Ensenada-Tijuana 3917, C.P. Ensenada 22860, Baja California, Mexico; 2 Departamento de Biología de la Conservación, Centro de Investigación Científica y de Educación Superior de Ensenada (CICESE), Ensenada 22860, Baja California, Mexico; 3 Facultad de Negocios Sostenibles, Universidad del Medio Ambiente, San Mateo Acatitlán, Valle de Bravo 51200, Estado de Mexico, Mexico

**Keywords:** *Capsicum annuum* var. *glabriusculum*, pavement cells, resistance to herbivores, *Spodoptera frugiperda*, trichomes, stomata, stomatal conductance

## Abstract

During domestication, lineages diverge phenotypically and genetically from wild relatives, particularly in preferred traits. In addition to evolutionary divergence in selected traits, other fitness-related traits that are unselected may change in concert. For instance, the selection of chili pepper fruits was not intended to change the structure and function of the leaf epidermis. Leaf stomata and trichome densities play a prominent role in regulating stomatal conductance and resistance to herbivores. Here, we assessed whether domestication affected leaf epidermis structure and function in *Capsicum annuum*. To do this, we compared leaf stomata and trichome densities in six cultivated varieties of Mexican *Capsicum annuum* and their wild relative. We measured stomatal conductance and resistance to herbivores. Resistance to (defense against) herbivores was measured as variation in the herbivory rate and larvae mortality of *Spodoptera frugiperda* fed with leaves of wild and cultivated plants. As expected, the different varieties displayed low divergence in stomatal density and conductance. Leaf trichome density was higher in the wild relative, but variation was not correlated with the herbivory rate. In contrast, a higher mortality rate of *S. frugiperda* larvae was recorded when fed with the wild relative and two varieties than larvae fed with four other varieties. Overall, although domestication did not aim at resistance to herbivores, this evolutionary process produced concerted changes in defensive traits.

## 1. Introduction

Domestication is an evolutionary process in which plant and animal species are consciously and unconsciously modified by humans through artificial selection [1,2,3]. An outcome of this process is that the selection of particular traits produces dramatic changes that clearly distinguish domesticated lineages from their wild relatives [4,5]. Domestication in plants is also accompanied by changes in the environment from wild to agricultural conditions, which may act as a selective factor over other phenotypic traits, favoring phenotypes that confer an advantage under new conditions [6,7,8]. However, some phenotypic traits could remain similar between wild and domesticated varieties because those phenotypic traits have physiological and ecological functions that are equally relevant for survival and reproduction in natural and in agronomic conditions [9,10,11]. A scenario such as this predicts that some traits will, and some will not, change under domestication, depending on their relevance for plant functioning. Although domestication produces ubiquitous divergence between cultivated and wild progenitors in selected traits, its effects on functional traits, which are not the target of selection, are scarce in the literature [11,12]. Therefore, to improve our knowledge of the adaptation of cultivars to agronomic conditions, it is necessary to examine the pattern of variation in those traits.

The leaf epidermis is the outermost layer of leaves that interacts with the external environment, and it comprises pavement cells, stomata, and trichomes [13]. The epidermal phenotype plays a critical role in the physiological and ecological aspects of wild plants and crops [14]. Among other functional roles, pavement cells and stomata actively participate in water regulation and gas exchange [15]. In turn, leaf trichomes protect plants from insects, thus acting as a functional barrier to reduce herbivore damage [16,17,18] while minimizing water losses by evapotranspiration and protecting the plant from UV radiation [19,20].

Studies of different plant species have shown that leaf epidermises can evolve under domestication [21]. The epidermises in cultivated varieties have diverged from those of their wild relatives in the type and number of cells implicated in the ecological functions of plants, such as reductions in the type and number of trichomes [22] or in the size or number of stomata or pavemented cells [23,24]. However, these modifications could be specific to some degree for each wild-cultivated system of plants. For example, Milla et al. [23] compared the size and number of stomata between the upper and lower epidermises of 24 wild-domesticated systems (pairs of plants), finding a significant reduction in stomata density in the lower epidermis and an increase in the size of stomata of the upper epidermis of domesticated leaves. Their results show a relationship between the number of leaf stomata in the upper epidermis and maximal stomatal conductance, indicating that the effects of domestication on epidermal traits were accompanied by consequences for the regulation of water evapotranspiration.

Domestication can reduce plant resistance to herbivores by affecting physical or chemical traits [9,25,26]. Evidence has demonstrated that, in most cases, domesticated varieties have a lower level of resistance than wild relatives [27,28,29]. Trichomes are among the resistance traits that can be modified during domestication [30]. The presence and number of trichomes can decrease abruptly in cultivars as compared to those in wild progenitors, such as tomato, sunflowers, and Chaya (*Cnidosculus aconitifolius*) [22,31,32]. This reduction in trichome density has been found to reduce the resistance of herbivores in cultivars. A study that compared 29 wild-cultivated plant systems found that the reduction in leaf trichomes was not accompanied by a cost in resistance for all species [33]. Their results indicate that the cost of the reduction in leaf trichomes that accompanies domestication depends on particular varieties. Therefore, to assess this idea, it is necessary to examine wild-cultivated plant systems, including more cultivars in the comparison, to better understand the consequences of domestication and the generalization of its effects on the functional traits of plants.

*Capsicum annuum* var. *glabriusculum* is the wild relative of most Mexican chili varieties cultivated in Mexico and several varieties domesticated abroad. Archeological registries suggest that *C. annuum* var. *glabriusculum* was domesticated in north and central Mexico in about 6400 B.P. [34,35], and further selection of fruits has resulted in about 60 varieties cultivated with contrasting fruit morphologies. The wild *C. annuum* var. *glabriusculum* populations are mostly found in tropical dry forests and tropical wet forests [36]. Northwest populations are exposed to stressful conditions of herbivory and water availability that affect plant fitness [37,38], whereas, in the south, the wild chili is often found in shady areas and is perhaps less prone to hydric stress compared to northwest populations [39]. In contrast, domestication of the cultivated varieties has occurred under beneficial conditions of water supply and under a lower exposure of plants to herbivores by using pesticides compared to wild relatives under natural conditions [40], relaxing the selective pressure of herbivores on resistance. Studies of wild and domesticated varieties of *Capsicum annuum* have documented notable differences in fruit size and morphology as a result of domestication [41]. Moreover, a recent study found a relationship between fruit size and the oviposition patterns of pepper weevils [27]. However, the phenotypic divergence of vegetative traits implicated in resistance and water regulation has received less attention. Two studies compared a single cultivated variety with its wild relative [23,33] but see [27], which limits our understanding of the broad spectrum of functional results expected in this group of plants [42].

Here, the aim of this study was to assess the impact of domestication on the epidermal traits implicated in water-use efficiency and resistance to herbivores in the wild-cultivated system *Capsicum annuum*. Stomatal density and conductance, as well as leaf trichome density and resistance to herbivores, were measured in six varieties of Mexican chili pepper and compared to its wild ancestor *C. annuum* var. *glabriusculum*. First, it was expected to find a higher density of stomata and higher water evapotranspiration in cultivated plants because cultivated plants have evolved under agronomic conditions, where they have been exposed to beneficial conditions of water availability in relation to wild relatives under natural conditions [43]. Likewise, a reduction in the density of leaf trichomes and a reduction in plant resistance to herbivores was expected in cultivated varieties as a consequence of plant domestication [27,28,29]. To assess the role of trichomes in resistance, variation in leaf trichome density was associated with the level of damage caused by a generalist herbivore *Spodoptera frugiperda.* This is the first study assessing how epidermal traits, critical to plant functioning, diverge between cultivated and wild varieties of *C. annuum*. These results show that plant resistance to herbivores has decreased in some cultivated varieties of *Capsicum annuum* highlighting the need to study differences in plant functioning and herbivore resistance in many varieties to better understand the outcomes and mechanisms of evolution under domestication in *C. annum* and other plants.

## 2. Results

### 2.1. Variation between Upper and Lower Epidermal Surfaces

**Figure 1:**
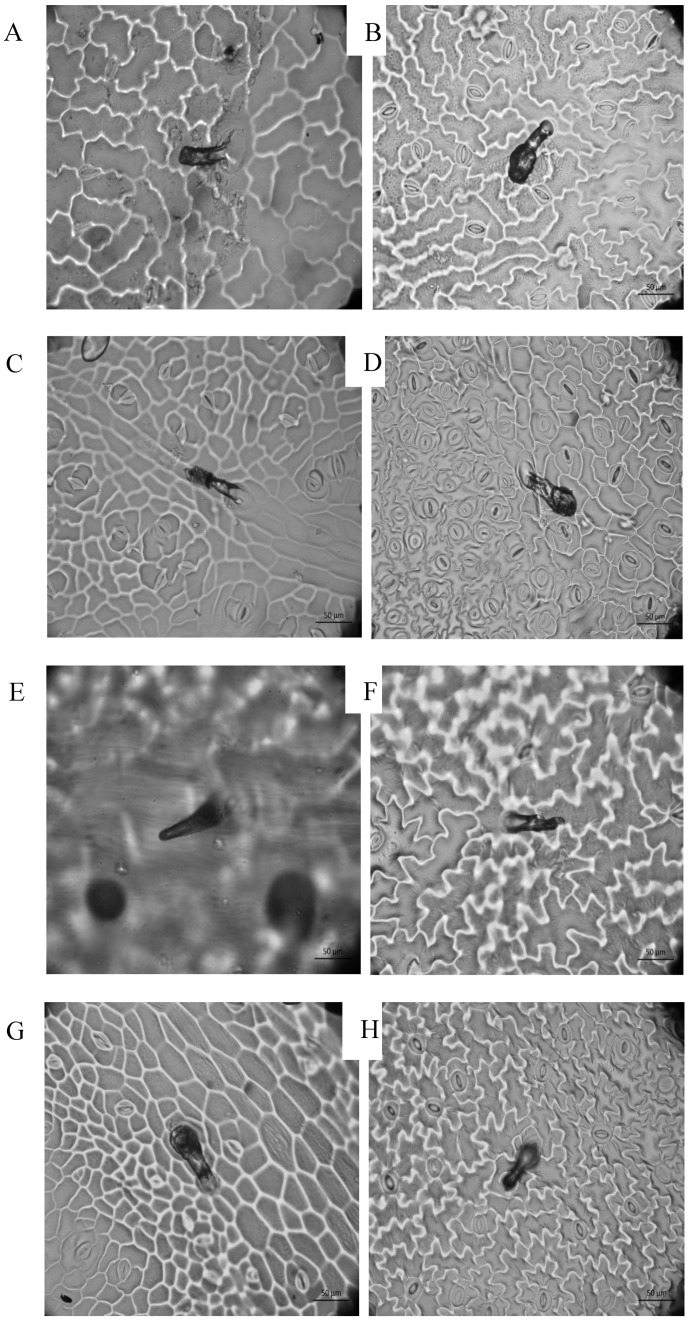
Epidermal cell imprints of domestic varieties (lower and upper sides) stomata and glandular trichomes in C and G. Chile de arbol (**A**,**B**), Güero (**C**,**D**), Jalapeño (**E**,**F**), and Pimiento (**G**,**H**) (40×).

The results show significant differences between varieties in five out of six epidermal traits (Table 1). Moreover, the leaf-side effect was significant for five out of six traits of density or cell size, indicating that the upper and lower epidermies differed in some cell types (Table 1 and Figure 1). The lower epidermis had a higher mean density and mean cell size than the upper epidermis (Figure 1). The covariate leaf area was only significant and positive for the number of pavement cells (Table 1). The mean stomata width was the only trait that did not show significant differences between varieties and between the upper and lower epidermis.

### 2.2. Stomatal Density and Size of the Upper and Lower Epidermis

The stomatal density of the lower and upper epidermises was significantly different between the chili plants (Table 2). The stomatal density of the lower epidermis differed between the wild population and two out of six cultivated varieties, YAHU and JAL. The mean stomatal densities of the YAHU and JAL varieties were 45% and 70% lower in comparison to the wild population (Tukey test _lower epidermis_: *α* = 0.05; Q = 3.304). In contrast, no differences in stomatal density were detected in the upper epidermis between the wild population and the cultivated varieties (Tukey test _upper epidermis_: *α* = 0.05, Q = 3.304).

The stomatal length was significantly different between the wild population and the cultivated varieties in the lower epidermis (Table 2 and Figure 1). In this case, the stomatal length of the wild population only differed significantly from that of the JAL variety, which had a 23% larger stomatal length than the wild variety (Figure 1). The stomatal length of the upper epidermis and the stomatal width of the upper and lower epidermises were not significantly different.

### 2.3. Stomatal Conductance

**Figure 2:**
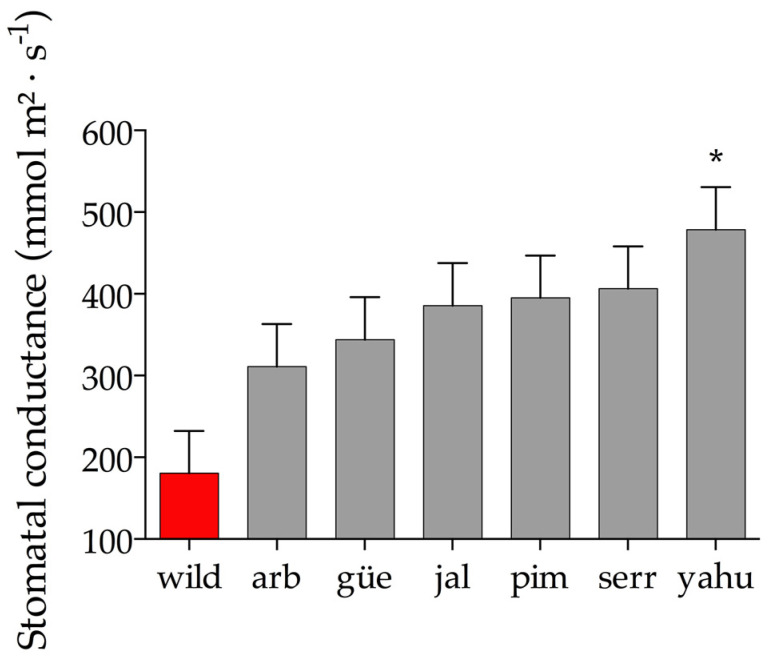
Univariate ANOVA was used to compare the the stomatal conductance of wild populations and cultivated varieties. Red bars indicate the mean and 1 ± SE for the wild variety. Gray bars indicate the mean and SE of the cultivated varieties. The asterisks indicate which cultivated variety significantly differs from the wild variety after Tukey’s HSD test (*α* = 0.05).

The stomatal conductance was significantly different among the different varieties (F_1,6_ = 3.28; *p* = 0.03; n = 21; R^2^ = 0.41). The wild population had the lowest stomatal conductance value (180.52 ± 51.82 SE), whereas the YAHU variety had the highest values (478.63 ± 51.82 SE), resulting in a difference of 94% in stomatal conductance (Figure 2. The correlation between stomatal conductance and density was not significant (*r* = −0.06; *p* = 0.8; n = 7).

### 2.4. Trichome Density in the Upper and Lower Epidermis

**Figure 3:**
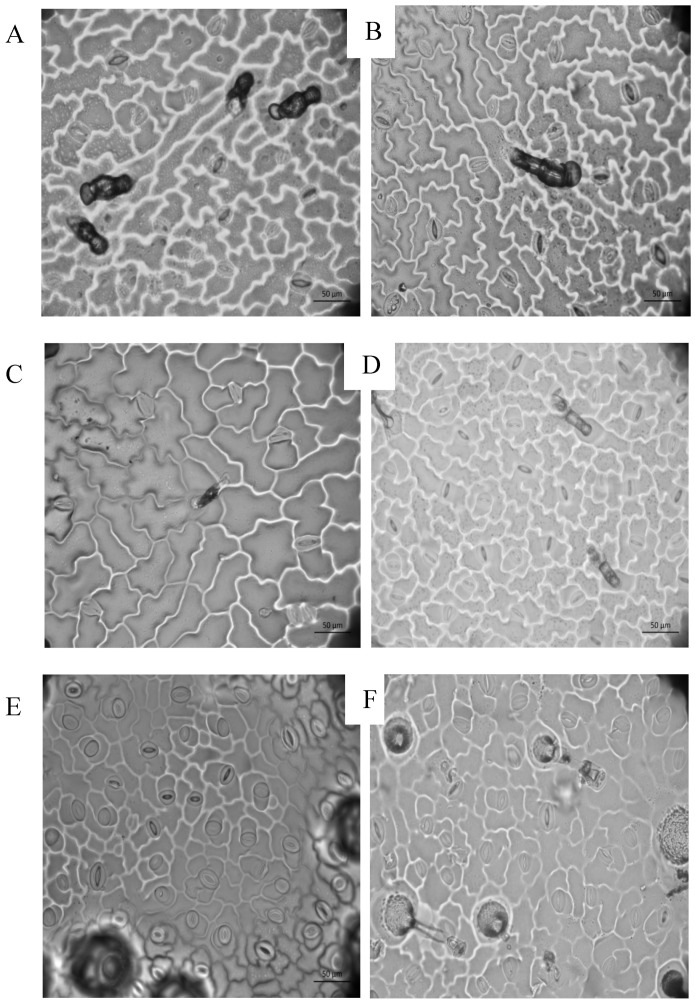
Epidermal cell imprints, stomata, glandular trichomes (**A**,**B**), and non-glandular trichomes (**E**) of domestic varieties (abaxial and adaxial), Serrano (**A**,**B**) and Yahualica (**C**,**D**), and a wild variety, San Javier (**E**,**F**) (40×).

**Figure 4:**
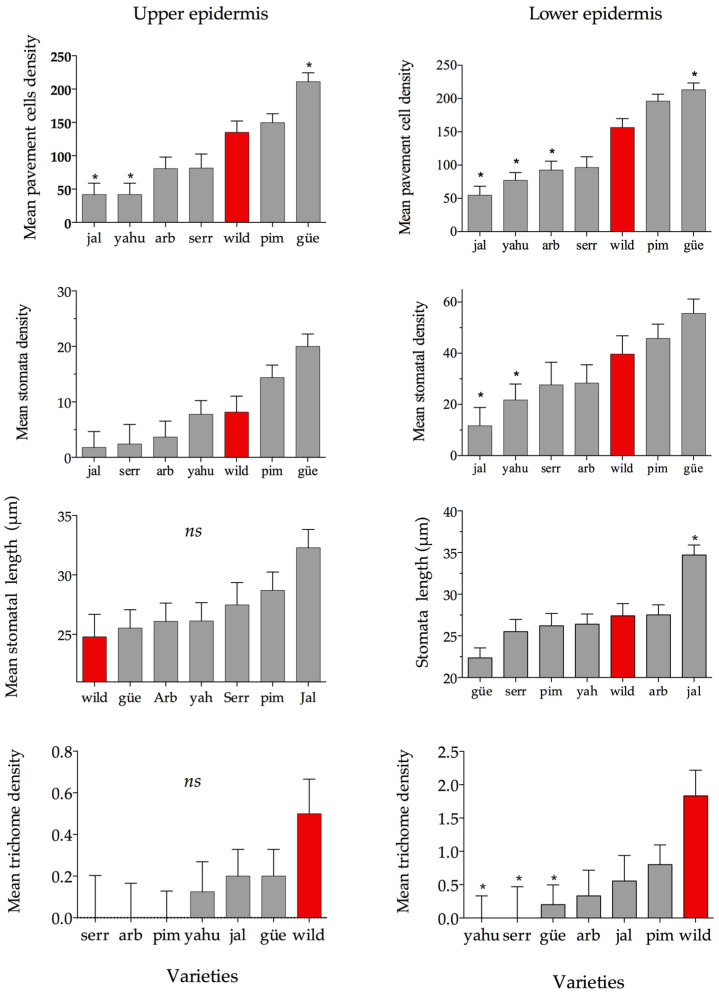
Independent ANOVA of the upper and lower epidermis for pavement density, stomatal density, length, and trichome density. Red bars indicate the mean and 1 ± SE for the wild variety. Gray bars indicate the mean and SE of the cultivated varieties. The asterisks indicate which cultivated variety significantly differs from the wild variety after Tukey’s HSD test (α =0.05).

The foliar trichomes identified in all tested varieties were glandular and non-glandular trichomes. Glandular trichomes were observed in the wild and all cultivated varieties (Figure 1C,G), whereas non-glandular trichomes were found in the wild population and in the cultivated JAL, SERR, and YAHU (Figure 3D,E). Leaf trichome density in the lower epidermis was significantly different between chilis and the wild population (Table 2 and Figure 4). The mean trichome density of the wild population was significantly higher than that of the domesticated GÜE, SERR, and YAHU varieties (Tukey test: *α* = 0.05; Q = 3.41). The trichome density of the upper epidermis was not significantly different among any of the varieties (Table 2).

### 2.5. Leaf Consumption Rate

**Figure 5:**
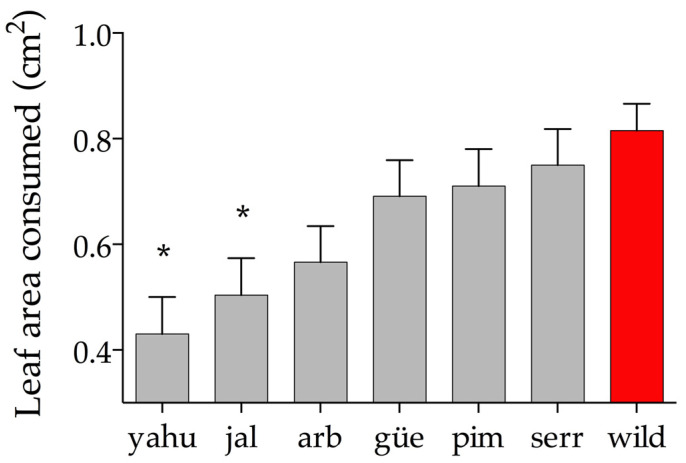
Univariate ANOVA was used to compare the leaf consumption rate of *Spodoptera frugiperda* of wild populations and cultivated varieties. Red bars indicate the mean and 1 ± SE for the wild variety. Gray bars indicate the mean and SE of the cultivated varieties. The asterisks indicate which cultivated variety significantly differs from the wild variety after Tukey’s HSD test (*α* = 0.05).

The leaf area consumed by *S. frugiperda* was significantly different among varieties and wild plants (F_1,6_ = 4.94; *p* < 0.02; n = 180; R^2^ = 0.08). Wild plants received the highest level of damage compared to cultivated varieties. The leaf area consumed by the wild variety was 43% higher than that of YAHU and 37% higher than that of the JAL varieties (Figure 5). The correlation between the consumption rate and the trichome density of the lower epidermis was not significant (r = 0.42; *p* = 0.3).

The larval survival of *S. frugiperda* was significantly different between cultivars and between cultivars and wild plants (*χ*^2^_L-R_ = 171.22; d.f. = 6; *p* < 0.0001; R^2^ = 0.65; n = 192). The lowest survival rate was found in larvae fed with leaf tissue of the wild population and two cultivated varieties (ARB and Yahu). In contrast, survival rates in larvae fed with tissue of other domesticated plants were higher, ranging from 100 to 95% for GÜE and PIM, and for the JAL and SERR varieties, survival was ~75%.

## 3. Discussion

This study is the first multi-variety assessment of the consequences of domestication, linking the morphology of the leaf epidermis and the function of *Capsicum annuum* (L.), one of the most important crop spices around the world. We included six cultivated varieties of *C. annuum* that are known to have been derived from their wild ancestor, *C. annuum* var. *glabriusculum*, to interpret the results following an evolutionary framework [34]. Domestication can produce abrupt changes, primarily increasing the size of selected traits [10]. However, this trend varies in magnitude, and in some crops, varieties show no changes in some traits even when they have relevant physiological and ecological roles in agricultural ecosystems.

This study found variation in the magnitude of the effect of domestication on epidermal phenotypes and their functions between wild populations and cultivated varieties of *Capsicum annuum*. The results reveal partial support for the effect of domestication in epidermal phenotypic traits and their function in *Capsicum annuum*. Stomatal densities showed great variation between varieties, but only two cultivated varieties differed from their wild relative. At the functional level, ample variability was found in stomatal conductance within all and the wild population, which is in line with the low differentiation in stomatal density and size. In relation to the effects of domestication on plant resistance, the wild *C. annuum* var. *glabriusculum* and two domesticated varieties had the strongest effects on herbivore survival, while four varieties caused lower or no herbivore mortality. We found higher trichome density in wild plants compared to cultivars. However, changes in trichome density did not show an association either with consumption rate by *S. frugiperda* or with herbivore survival. Overall, the patterns observed in this study show the complex effects of domestication on the leaf epidermis of *C. annuum*, suggesting that domestication may be moderate in traits that are not consciously selected.

Stomatal density and size can increase during domestication because cultivated plants are exposed to new environmental conditions, such as high water availability [44]. In particular, the densities and sizes of stomata are expected to increase during domestication if water losses are not traded off with the rate of CO_2_ diffusion to the interior of the leaf [45]. We found significant variations between the wild population and varieties in terms of stomatal density and length. However, differences in stomatal density between the wild and cultivated varieties were found only in the lower epidermis. This low-level differentiation can explain the similarities in evapotranspiration rates found between the wild and cultivated plants in this study. Although the wild variety showed the lowest rate of evapotranspiration, it only had a significant difference from one cultivated variety (the YAHU variety).

The low level of differentiation observed in stomatal density and size (i.e., length) is not rare. In agreement with our results, previous studies have found that stomatal density and length do not show a high degree of divergence between wild and cultivated varieties in the common bean, *Phaseolus vulgaris* (Fabaceae) [24]. Moreover, an assessment of 24 species found that stomatal features showed low differentiation between wild and cultivated relatives [23]. These moderate differences in the density and size of stomata between the cultivated varieties and their wild ancestor explain the absence of a correlation between stomatal conductance and stomatal density [46]. Thus, the low degree of divergence in density and size between the cultivated varieties and their wild relative can explain the similitude in stomatal conductance observed in this study as well. In this case, this evidence suggests that the agronomic conditions of water availability did not promote an increase in stomatal traits implicated in ecological functions.

The effects of domestication were more evident in leaf trichomes than in the other epidermal cells (stomatal and pavemented cells). In this case, the wild plants had significantly more trichomes than the plants of three out of six cultivated varieties in the lower epidermis. As expected, the wild population had the highest density of trichomes, whereas the most dramatic change was that leaf trichomes were not detected in some cultivated varieties. Surprisingly, variation in trichome density did not correlate with the herbivory rate. Contrarily, the herbivory rates of *S. frugiperda* were higher in the leaf disks of wild plants in relation to domesticated plants. Given that wild plants had the highest trichome density, this study suggests that leaf trichomes did not effectively obstruct feeding by *S. frugiperda* larvae [47]. Previous studies have found similar evidence suggesting that changes in leaf trichome density associated with domestication did not explain the resistance to herbivores [33]. Thus, even when leaf trichomes seemed to not have an adaptive role as a component of resistance to herbivores, it remains to be tested whether trichomes provide defense for other herbivore species.

One objective of this study was to assess whether domestication reduces resistance to herbivores. Variation in mortality rate between wild and cultivated plants found in this study supports the hypothesis that domestication affects plant resistance to herbivores in *Capsicum annuum*. We found that *S. frugiperda* larvae exposed to a leaf-based diet of wild populations and two cultivars caused a dramatic survival reduction in *S. frugiperda*. In contrast, the leaf tissue of the other four cultivars had a lower impact on insect mortality. These results are comparable with evidence from a recent meta-analysis that found that insects fed with the leaf tissue of wild plants have higher mortality in relation to domesticated ones [48]. Specifically, in *Capsicum annuum*, previous studies have found similar results, supporting the notion that domestication reduces the level of resistance to herbivores as a function of insect mortality [49]. For instance, when resistance to herbivores in a wild population (i.e., Tapachula accession, not included in this study) and cultivated poblano and serrano varieties of *C. annuum* were compared, the mortality rates of *S. frugiperda* fed wild tissue were higher than those fed domesticated varieties [49]. Although resistance to herbivores decreased with domestication in four varieties analyzed in this study, wild and two cultivated chili varieties had a similar impact on insect mortality, suggesting that the cost of domestication is not equal in all species. Thus, domestication effects on plant defense largely depend on the variety and its evolutionary history, and they also need to be assessed on insects.

One caveat in this study is that we did not measure a biochemical component of resistance to herbivores. Other studies have found that leaf polyphenols affect *Spodoptera* performance [50,51], indicating the relevance of these types of compounds in resistance to herbivores. In our case, secondary metabolites, such as polyphenols, could explain resistance to herbivores, and domestication can affect chemical resistance to herbivores in Mexican varieties of *Capsicum annuum*. Further metabolomic studies are required to compare and identify the type and number of chemical compounds responsible for the mortality of the herbivore *S. frugiperda* in wild and cultivated varieties of *C. annuum*.

This is among the few studies that have analyzed the changes in epidermal cells in different cultivated varieties as well as their functional implications. The lack of differences in the density of stomata and their conductance indicates that this trait has not been abruptly affected during the course of domestication. Second, wild plants produced significantly more trichomes than cultivars, but the trichomes did not obstruct herbivore feeding, as expected for a trait with a defensive role. In spite of this, the high survival rate of *S. frugiperda* fed with cultivated tissue indicated that domestication can have detrimental effects on plant resistance [48], but it seems that changes are occurring in secondary compounds. Traits related to water-use efficiency/CO_2_ uptake and resistance to herbivores can show ample variation with no fitness cost for domesticated plants [23,43,52] possibly as a result of the relaxed selection occurring under agronomic conditions (i.e., irrigation and pesticide application). To examine the cost of domestication, further studies should expose cultivars to simulate the natural conditions of water stress and/or different levels of herbivores [53]. This will help clarify how cultivars depart from their ancestor and whether small phenotypic modifications [54], such as stomata and trichome density, associated with domestication in *Capsicum annuum* are relevant in natural environments [55].

## 4. Conclusions

Our results demonstrate for the first time that domestication of *C. annuum* is accompanied by changes in epidermal traits implicated in critical functional traits. We confirmed that domestication has detrimental effects on resistance to herbivores in some varieties, but the differences in leaf trichomes failed to explain variation in resistance to herbivores. Likewise, water evapotranspiration and stomata did not show dramatic changes. These findings thus have implications, which we discuss in more detail above, for understanding both the broad spectrum of functional responses expected under domestication in this group of plants and the variable consequences of domestication.

## 5. Materials and Methods

### 5.1. Plant Materials

For the experiment, we used seeds from six cultivated varieties and the wild relative *Capsicum annumm* var. *glabriusculum*. Seeds of the wild *C. annuum* var. *glabriusculum* were collected during 2016 and 2017 from different natural populations and stored in a germplasm collection. To examine epidermal features, we used wild seeds collected from San Javier, Sonora, located in northwestern Mexico. Furthermore, to measure water-use efficiency and plant resistance, we also included seeds collected from two more wild populations, namely Veracruz and Tapachula, which grow under more humid conditions. Those populations were included to cover a broad spectrum of variation in stomatal conductance, water-use efficiency, and resistance to herbivores (Table 1). Seeds of the six cultivated varieties used were obtained from commercial stocks from a local market brand, Rancho Los Molinos (Mexico). The following cultivated varieties were used: *Capsicum annuum*, called “árbol” (ARB), “jalapeño” (JAL), “serrano” (SERR), “yahualica” (YAHU), “güero’’ (GÜE), and “morrón” (PIM). These varieties were randomly chosen, but we took care to include varieties for whom *C. annuum var. glabriusculum* is the common ancestor [54,55].

### 5.2. Plant Growth

We sowed 50 seeds per variety in germination trays filled with commercial soil (Berger BM2). All trays were placed in a germination room with a controlled photoperiod with a 14:10 h nightlight under a temperature of 25 ± 2 °C. Seedlings with four well-expanded leaves were transplanted to 4 L plastic pots filled with substrate (Berger BM2). A total of 15 seedlings per variety were transplanted, resulting in 105 plants from the San Javier wild variety and the six cultivated varieties. The plants were randomly distributed on six benches within a greenhouse to avoid shading between plants. During the experiment, fertilization consisted of a weekly application of 50 mL of solution containing 2 g × L^−^^1^ N-P-K (19-19-19) on the soil of each plant. All plants were watered ad libitum to avoid the potential influence of hydric stress on the leaf epidermis. In addition, the instructions of the seed producers indicated that the plants had to be very well irrigated.

### 5.3. Epidermal Impressions

To examine variations in the leaf epidermis, we collected leaves from a subsample of three plants per variety brought to the laboratory from the greenhouse. From each plant, we collected five mature leaves from the middle part of the stems of each individual plant. We obtained 400 epidermal impressions from the abaxial and adaxial surfaces of the sampled leaf. Epidermal impressions were made using the nail polish method. Briefly, we applied a film of clear nail polish to the leaf surface between the second and third secondary veins, avoiding the main and secondary veins. The nail polish was left to dry for 3 min, and then we placed a piece of transparent adhesive tape on the area covered with the nail polish. We pressed the adhesive tape evenly for 1 min using a piece of cotton rag. The adhesive tape was removed using fine scissors, and the sample was placed on a slide labeled with the name of the variety and the sample number.

### 5.4. Cell Counts and Stomatal Measurements

To obtain the number and measurements of epidermal cells, we digitized the upper and lower epidermises on a sample of 3–5 leaves per plant using a light optical microscope Primo star equipped with an ICc5 digital camera (Zeiss, Germany). A 40× magnification lens was used to obtain digital images, resulting in a visual area of 2.38 mm^2^ for each sample. We counted all pavement cells, trichomes, and stomata observed in each digital image, excluding incomplete cells at the margins of images. Stomata length and width measurements were obtained from three closed stomata randomly chosen from the samples. Stoma length was measured as the distance from the upper to the lower tip of a guard cell. In turn, the stoma width was measured as the distance between the outer extremes of the two guard cells in the middle of each stoma. Cell counts and stomatal measurements were conducted using Zen lite software (Zeiss 2012).

### 5.5. Stomatal Conductance

Stomatal conductance was measured in a subsample of three plants per variety. Measurements were taken on a random sample of three mature leaves located in the middle part of the stem of each plant. Stomatal conductance was obtained on the upper side of the leaves using a leaf porometer (SC-1 model, Decagon Services) between 11:30 am and 12:00 pm. Plants from each variety were randomly distributed in the greenhouse. All plants were well irrigated for two days before measurements were conducted.

### 5.6. Insects

*Spodoptera frugiperda* eggs were obtained from the Laboratorio de Control Biológico, ECOSUR-Tapachula. The eggs were allowed to hatch in Petri dishes (90 mm × 15 mm) on moist paper in a growth room (L:D 14:10 h; 26° C; 65% RH). Larvae were then individually moved to small plastic containers in the same growth room on a corn-based diet. Third-instar and fourth-instar larvae were used for both insect consumption rates and survival tests.

### 5.7. Insect Consumption Rate

To determine whether domestication influences insect consumption rates, third- and fourth-instar *S. frugiperda* larvae were given leaf disks from each of the six cultivated varieties and from each wild population. Plastic Petri dishes with a 3 cm diameter × 2 cm depth were used as arenas. Leaves were randomly removed from cultivated and wild plants in the greenhouse and brought to the laboratory, where a hole punch (1.96 cm^2^ diameter) was used to cut the leaf disks. One leaf disk was placed into each Petri dish, and one larva was placed into a Petri dish and allowed to feed on leaf disks for 1 h in the growth room (see above). One hour was determined to be a reasonable length of time to assess consumption rates of herbivores but short enough to prevent insects from consuming all of the leaf disks from one treatment. A total of 24 larvae were assigned to be fed per domesticated variety or wild population, resulting in 216 larvae. When a larva did not start to eat within the first 20 min, it was excluded from the experiment. This resulted in 189 larvae. At the end of the experiment, the remaining leaf disks were removed from the Petri dish and digitized to obtain the remaining leaf disk areas for each cultivated variety and wild population. The remaining leaf areas were determined using the imaging program WinFolia (Regent Instruments, USA). The remaining leaf disk areas were subtracted from the original area of the leaf disks to determine the area consumed. We did not replace larvae because of the short time elapsed in this experiment.

### 5.8. Survival assay of Spodoptera frugiperda

To test whether domestication influenced insect survival, the larvae of *Spodoptera frugiperda* were allowed to feed on leaves from cultivated and wild plants from each of the six cultivated lines and the three wild accessions. The experiment began with a sample of 216 larvae, but larvae that did not start to eat after 4 h were excluded from the experiment, resulting in a sample size of 192. Each individual larva was placed in a Petri dish fed with a 1.5 cm^2^ leaf tissue disk of only one variety until death or pupation in the growth room (see above). The leaf disks were changed every 12 h to maintain fresh tissue. This time was determined to be a reasonable length of time to maintain fresh tissue and was short enough to prevent insects from consuming all of the leaf disks and starving. Survival (1) and mortality (0) were registered daily until the last larva died or reached the pupa stage. All larvae fed the leaf tissue of wild chili died before reaching the pupa stage.

### 5.9. Statistical Analyses

First, an analysis of variance (ANOVA) of the leaf size was conducted, including the variety as a fixed effect to compare the leaf size between the wild populations and cultivated varieties. Second, a univariate analysis of covariance (ANCOVA) was conducted to compare the density and size of epidermal cells among the wild populations and cultivated varieties, including the following variables: (i) pavement cell density, (ii) density of stomata, (iii) stomatal length, (iv) stomatal width, and (v) trichome density. The ANCOVA included the effects of variety, leaf side (i.e., the upper and lower epidermises), and the variety × leaf size interaction. For these analyses, data on the upper and lower epidermises were pooled. When leaf size was not significant for a specific trait, it was removed from its respective ANCOVA. Furthermore, we performed Tukey’s test to assess the significant differences between the wild population and cultivated varieties.

Because the number of cells differing between the upper and lower epidermises was statistically different, we conducted independent ANOVAs for the upper and lower epidermis for the same response variables mentioned above. This model only included the effect of variety as a fixed effect. We performed Tukey’s test comparisons to assess the significant differences between the wild population and cultivated varieties.

To examine the differences in stomatal conductance and consumption rate by herbivores, we conducted an ANOVA for each variable, including variety as a fixed effect. Survival among larvae was analyzed with a logistic ANOVA, including variety as a fixed effect. Data of larvae fed wild chili plants were pooled because the consumption rate (F = 0.99; g.l. = 5; *p* = 0.4) and survival were not different between populations.

To examine the relationship between a trait and its functional role, individual Pearson’s correlation analyses were used for pairs of variables. One correlation was estimated for stomatal conductance vs. stomatal density, and another correlation was conducted for consumption rate vs. trichome density. These correlations were performed using the mean values per variety calculated prior to the analysis. Data analysis was performed using JMP software v. 10.0 (SAS Cary, NC, USA).

## Figures and Tables

**Table 1:** Univariate ANOVA of cell densities and sizes of leaf epidermis of the wild *Capsicum annuum* var. *glabriusculum* and six cultivated varieties of Mexican *C. annum*. The sample size for cell density counts was 25 plants, and for cell size measurements, it was 19 plants. *p* < 0.05 indicates significant differences.

Trait	Source of Variation	d.f.	m.s.	F	*p*
Pavemented cell density	Variety	6	143,410.06	38.86	<0.0001
	Leaf side	1	4192.23	6.82	0.0126
	Leaf area	1	3341.12	5.43	0.0248
Stomata density	Variety	6	5377.80	8.84	<0.0001
	Leaf side	1	8183.47	80.71	<0.0001
Trichome density	Variety	6	5.60	3.19	0.0114
	Leaf side	1	2.07	7.06	0.0111
Stomata width	Variety	6	23.31	1.05	0.4
	Leaf side	1	69.38	18.80	0.0002
Stomata length	Variety	6	321.71	8.80	<0.0001
	Leaf side	1	0.26	0.04	0.8

**Table 2:** Univariate ANOVA to compare the density and size of the upper and lower epidermis of the wild *Capsicum annuum* var. *glabriusculum* and six Mexican cultivated varieties.

Leaf Side	Trait	Source of Variation	d.f	s.s	F	*p*
Upper epidermis	Pavemented cell density	Variety	6	84,320.8	15.72	<0.0001
	Stomata density	Variety	6	1065.22	7.15	0.0005
	Trichome density	Variety	6	0.65	1.31	0.3
	Stomata width	Variety	6	20.015	0.92	0.5
	Stomata length	Variety	6	110.57	2.43	0.08
Lower epidermis	Pavemented cell density	Variety	6	91,355.631	28.6	<0.0001
	Stomata density	Variety	6	5339.69	5.75	0.0017
	Trichome density	Variety	6	7.8	2.94	0.035
	Stomata width	Variety	6	15.83	0.57	0.7
	Stomata length	Variety	6	248.89	9.19	0.0007

## Data Availability

The data presented in this study are available on request from the corresponding author.
